# The critical role of RasGRP4 in the growth of diffuse large B cell lymphoma

**DOI:** 10.1186/s12964-019-0415-6

**Published:** 2019-08-13

**Authors:** Lin Zhu, Chunyan Xia, Lin Wu, Yuxuan Zhang, Junling Liu, Yinan Chen, Jing Liu, Yongxin Xiao, Kai Nie, Liyu Huang, Ning Qu, Hong Yu

**Affiliations:** 1 Shanghai Chest Hospital Affiliated to Shanghai Jiao Tong University, No. 241 West Huaihai Road, Shanghai, 200030 China; 2 Shanghai Changzheng Hospital Affiliated to the Second Military Medical University, Shanghai, China; 3 Shanghai General Hospital Affiliated to Shanghai Jiaotong University, Shanghai, China; 4 School of Pharmacy, Queen’s University Belfast Medical Biology Centre, Belfast, UK; 5 School of Medicine, Shanghai Jiao Tong University, Shanghai, China; 6 Fudan University Shanghai Cancer Center, Shanghai, China

**Keywords:** RasGRP4, DLBCL, MAPK, Proliferation, Prognosis

## Abstract

**Background:**

This study aimed to confirm that blocking RasGRP4 can effectively slow down the growth of DLBCL both in vitro and in vivo and ascertain the role of RasGRP4 in the prognosis of DLBCL clinically.

**Methods:**

RasGRP4 expression levels were examined in benign tissues and lymphomas. In order to verify somatic mutation in RasGRP4 gene, cDNA sequencing was performed in DLBCL patients. RasGRP4-dependent cell proliferation, mitochondrial membrane potential, oxidative stress levels and signaling pathway changes were measured by knockdown of RasGRP4. Tumor growth was monitored in xenografted lymphoma model. Clinical data were collected to confirm the role of RasGRP4 in DLBCL.

**Results:**

RasGRP4 expression was significantly elevated in DLBCL while no somatic mutations were detected of this gene in DLBCL patients. Decreased RasGRP4 significantly inhibited cell proliferation by simultaneously reducing mitosis and promoting apoptosis and increased the oxidative stress levels. Mechanistically, reduced expression of RasGRP4 decreased ERK while increased JNK expression in SUDHL-4 cells. Knockdown of RasGRP4 also significantly inhibited tumor formation in vivo. Furthermore, RasGRP4 expression levels were significantly higher in patients with larger DLBCL lesions (*P* = 0.0004), high-risk international prognostic index score groups (*P* = 0.0042), and its expression was positively correlated with maximum standardized uptake value in DLBCL (*P* = 0.0004).

**Conclusions:**

These findings indicate the oncogenic role of RasGRP4 in DLBCL, suggesting it as a prognostic biomarker and potential therapeutic target in DLBCL.

**Electronic supplementary material:**

The online version of this article (10.1186/s12964-019-0415-6) contains supplementary material, which is available to authorized users.

## Background

Diffuse large B cell lymphoma (DLBCL) is one of the most common subtypes of non-Hodgkin lymphoma, accounting for 30% of new lymphoma cases annually [1, 2]. The median survival of patients with DLBCL is less than 1 year if left untreated [3, 4]. Although improvement in overall survival rates for DLBCL has been accomplished with the current recommended therapy, 30–40% of cases still relapsed after treatment [5–7].

Ras and the downstream mitogen-activated protein kinase (MAPK) family, which consists of extracellular signal-regulated kinase (ERK), c-Jun N-terminal kinase (JNK), and p38 MAPK, are implicated in B cell lymphomas and are considered promising therapeutic targets [8–10]. However, little is known regarding the involvement of these molecules that are directly upstream of these pathways.

Ras guanine nucleotide-releasing protein (RasGRP) family members are guanine nucleotide exchange factors that specifically control the exchange of GDP for GTP on the small GTPase Ras. These ultimately lead to downstream MAPK activation in many cellular processes [11–14]. However, whether the RasGRP molecules also have similar roles in lymphoma remains to be elucidated. RasGRPs have been proved to play some specific role in many kinds of lymphocytes, and among the RasGRP family members, RasGRP4 has been shown to be substantially different from other family members. It is highly expressed in mast cells, developing T cells, neutrophils and monocytes [15–17]. Previous research on RasGRP4 has mainly focused on mast cells and it has been proved to play an important role in the development and function of mast cells [18].

For the first time to our knowledge, we report significantly higher expression of RasGRP4 in DLBCL tumors compared to normal control tissues. Furthermore, we verified RasGRP4 as the most highly expressed molecule in DLBCL relative to other RasGRP family members.

Hence, in this study, we investigated the expression and location of RasGRP4 in B cell lymphoma, and showed significant overexpression of RasGRP4, especially in DLBCL. We report that reduction of RasGRP4 expression in DLBCL cells led to significant inhibition of cell proliferation in vitro and suppressed tumor growth in a nude mouse model in vivo by modulating the MAPK-associated signaling pathway. Moreover, elevated RasGRP4 expression was associated with larger tumor lesion size and multiple prognostic risk factors in DLBCL patients. These data suggest that RasGRP4 may play an important role in DLBCL oncogenesis, and the expression of RasGRP4 may act as an independent prognostic factor in DLBCL outcome.

## Materials and methods

### Human DLBCL tissue and benign tissue samples

Paraffin sections of human DLBCL tissues from randomly and anonymously selected patients were provided by Dongfang Hospital, Shanghai General Hospital, Shanghai Changzheng Hospital and Shanghai Chest Hospital. Benign human tissues were obtained from reactive lymph nodes of patients who underwent lymph node dissection with a benign or inflammatory pathological diagnosis. Fresh DLBCL tissues and non-neoplastic lymphoid tissues were collected immediately after surgical resection at Dongfang Hospital, Shanghai Changzheng Hospital and Fudan University Shanghai Cancer Center. Normal B cells or activated B cells were obtained from reactive or normal lymph nodes. Cells were disrupted by a cell strainer, washed with phosphate-buffered saline (PBS) at 4 °C, and centrifuged for 15 min at 4 °C to collect cells. All the cases were pathologically confirmed to have DLBCL, and the diagnoses of all tissue samples were verified by two pathologists.

### Immunohistochemistry

Expression of RasGRP4 protein levels was measured using immunohistochemistry (IHC). Paraffin sections (5 μm) of human DLBCL tissues and benign tissues were incubated at 60 °C for 15 min, deparaffinized with xylene, and rehydrated by graded alcohol. The slides were then incubated with 3% hydrogen peroxide and 0.2% Triton X-100. Antigen retrieval was performed at 95 °C for 30 min (the sections were placed in 0.01 M citrate buffer [pH 6.0]), then washed with PBS 3 times, followed by incubation with normal goat serum (Jackson Laboratory, 005–000-121, 1:200, final volume required for full coverage of specimens was based on the size of the sample) for 2 h. After that, the slides were incubated overnight with rabbit anti-human RasGRP4 antibody (Abcam, ab96293, 1:200) at 4 °C. On the next day, the slides were incubated with a biotinylated goat anti-rabbit IgG antibody (Jackson Laboratory, 111–035-003, 1:200, 30 min) and visualized with a streptavidin–biotin–peroxidase reaction (Vector Laboratories, 1:1, 30 min). After colorimetric reaction with 3,3′-diaminobenzidine solution (Vector Laboratories, 1:50, 3 min), the slides were dehydrated and mounted. Representative images were obtained with a Nikon ECLIPSE Ni microscope with color camera, and the expression of RasGRP4 protein was determined by measuring the average optical density (AOD) using Image-Pro Plus 6.0 software.

### Cell extraction and western blotting

Cells and tissues were disrupted by a cell strainer (BD Biosciences, 70 μm), washed with PBS, then collected by centrifugation for 15 min at 4 °C and lysed with radioimmunoprecipitation assay buffer (500 μl). Samples were centrifuged for 15 min at 4 °C and the supernatant was collected. Equal amounts of protein (35 μg) from the cell extracts were resolved in sodium dodecyl sulfate-polyacrylamide gel electrophoresis and then transferred to polyvinylidene fluoride (membranes. The membranes were blocked with 5% skim milk for 2 h and then incubated with primary antibodies against RasGRP4 (Abcam, ab96293), glyceraldehyde-3-phosphate dehydrogenase (GAPDH; Kangcheng, KC-5G4, 1:1000), phospho-ERK1/2 (Cell Signaling Technology, D13.14.4E, 1:100), ERK1/2 (Cell Signaling Technology, L34F12, 1:100), phospho-ERK5 (Cell Signaling Technology, 3371, 1:100), ERK5 (Cell Signaling Technology, D23E9, 1:100), phospho-P38 (Cell Signaling Technology, 9211 s, 1:100), P38 (Cell Signaling Technology, 9212, 1:100), phospho-JNK (Cell Signaling Technology, 81E11, 1:100), and JNK (Cell Signaling Technology, 56G8, 1:100), caspase-3 and cleaved capase-3 (ProteinTech, 19,677–1-1AP, 1:100) at 4 °C overnight. After washing with TBST, the membrane was incubated with horseradish peroxidase-conjugated secondary antibody (CST, 7074, 1:500; Abcam, ab136815, 1:500) for another 2 h at room temperature. The membranes were then visualized with an enhanced chemiluminescence system (GE Healthcare Life Sciences) according to the manufacturer’s protocol. The optical density was quantified by ImageJ software with FracLac plugin Java 1.6 (National Institutes of Health).

### Cell sorting and detection of protein expression

Reactive or normal lymph nodes were disrupted by a cell strainer, washed with PBS, and collected by centrifugation for 15 min at 4 °C. Cells were resuspended in 500 μl PBS and incubated with PE anti-human CD19 antibody (Biolegend, 302,208, 1:100, 1 h at 4 °C) for sorting of normal or activated B cells from the mixture using a FACSAria cell sorter (BD Biosciences). Cells were fixed in 4% paraformaldehyde (15 ml) for 30 min, permeabilized with 0.1% Triton X-100 (10 ml, 10 min), and incubated with RasGRP4 antibody (Abcam, ab96293, 1:200, 30 min) at 4 °C. Subsequently, RasGRP4 protein expression in lymphoma cell lines and in normal or activated B cells was detected by flow cytometry with a FACSCalibur instrument (BD Biosciences).

### Quantitative real-time PCR (qRT-PCR)

Total RNA was purified using TRIzol reagent (BD Biosciences) according to the manufacturer’s instructions. cDNA was prepared using a reverse transcription kit (Takara). qPCR was carried out using SYBR premix EX Taq (Takara) and then run on an ABI 7500 PCR instrument (Applied Biosystems). GAPDH was used as an internal control for gene expression normalization. The sequences of primers used to detect RasGRP1–4 gene expression are presented in Table 1.

**Table 1:** Primer sequences for RasGRP1–4 in qRT-PCR

Gene	Forward	Reverse
RasGRP1	5′- AGGAGTTACATTGCCGCCTGAT-3′	5′-CGTTTCTTGCTGGTATTTGATT-3′
RasGRP2	5′-TCCTGGAGCGGTTCATCT-3′	5′- CACTGCCATCAGCGTGTT-3′
RasGRP3	5′- CGCTTTCCCTGGACCTCTAT-3	5′- TGTGCTTGTTGATGACCGTG-3′
RasGRP4	5′-AGACCAGGTGAAGGTAGAATGT-3′	5′- TCCAGGGATAGCGTGTAGG-3′
GAPDH	5′- CTCAAGGGCATCCTGGGCTA-3	5′- ATGAGGTCCACCACCCTGTT-3′

### Extraction of nucleic acids and sequencing

Total RNA was extracted from samples of 4 patients using the Trizol reagent (Life Technologies) according to the manufacturer’s instructions. and cDNA synthesis was performed using the TransScript All-in-One First-Strand cDNA Synthesis SuperMix for qPCR (Transgen Biotech). RasGRP4 was amplificated using transtaq-T pcr superMIX (Transgen Biotech). The primers were as follows: RasGRP4 sense, 5′-ATGAACAGAAAAGACAGTAAGAGGAAGTCC-3′ and antisense 5′-CTAGGAATCCAGCTTGGAGGATGC-3′. The purified PCR products were cloned into pESI-T vector using Hieff Clone® Zero TOPO-TA Cloning Kit (Yeasen).10 monoclones of each patient were sequecing with the following primer, Forward primer 5′-TGTAAAACGACGGCCAGT-3′ and reverse primer 5′-CAGGAAACAGCTATGACC-3′.

### Cell culture and transduction

SUDHL-4 cells (human DLBCL cells; American Type Culture Collection) and Raji human Burkitt lymphoma cells (American Type Culture Collection) were cultured in RPMI-1640 (basal medium) supplemented with 10% fetal bovine serum (Gibco), 100 U/ml penicillin, and 100 U/ml streptomycin in an incubator at 37 °C in 5% CO_2_.

Lentiviral plasmids encoding the RasGRP4-targeting short hairpin RNA (shRNA) and a green fluorescent protein (GFP) reporter were purchased from Asia-Vector Biotechnology Co. Ltd. (Shanghai). The lentiviral vectors containing scrambled shRNA sequences were used as negative controls (NCs). The sequence for RasGRP4 shRNA-2, which was determined to be the most efficient shRNA used in the present study, was 5′-GCATCCTCCAAGCTGGATT-3′, and the sequence of control shRNA was 5′-CCTAAGGTTAAGTCGCCCTCGCTCGAGCGAGGGCGACTTAACCTTAGGTTTTT-3′. SUDHL-4 cells were infected with RasGRP4 shRNA and NC lentiviral vectors, and GFP expression was assessed by fluorescence microscopy after 48 and 72 h of transfection.

### Cell viability assay

Cells infected with RasGRP4 shRNA and NC lentiviral vectors were plated into 96-well plates (25,000 cells per well) in triplicate. Cell viability was determined by a Cell Counting Kit-8 (Dojindo) daily for one week after transfection. The absorbance was read at 450 nm using a spectrophotometer.

### Cell cycle analyses

After 72 h of transfection, the cells were harvested and fixed in 5 ml ice-cold 70% ethanol at 4 °C overnight. The fixed cells were washed and resuspended in 500 μl ice-cold PBS, and then incubated with 5 μl propidium iodide (PI) and RNase in the dark for 30 min at 37 °C. The stained cells were analyzed by a FACSCalibur flow cytometer.

### Apoptosis analysis

Cell apoptosis was examined by flow cytometry after 72 h of transfection. Cells were washed with 5 ml PBS 3 times and resuspended in 500 μl ice-cold PBS, then stained with 5 μl annexin V-APC (BD Biosciences, 550,475, 1:100, 10 min) in the dark. The cells were stained with 5 μl PI (BD Biosciences, 550,475) for 30 s according to the manufacturer’s recommendations. Annexin V^+^/PI^−^ (early apoptotic) cells and annexin V^+^/PI^+^ (late apoptotic) were quantified in the infected cells with GFP based on the frequency of fluorescently labeled cells. Ten replicates of shRNA- and NC-transfected cells were performed, and statistical significance was assessed by two-sample *t* test.

### Measurement of mitochondrial membrane potential

JC-1 probe (YEASEN, 40705ES03) was used to detect mitochondrial depolarization. JC-1 staining buffer (5 mg/ml) was diluted with pre-warmed culture medium to the desired working solution concentration (10 μg/ml) and was thoroughly mixed. The cells in 6-well plates were collected and washed in PBS and then incubated in diluted JC-1 buffer (1 ml) for 30 min at 37 °C. Then, the cells were harvested and washed with PBS 2 times at room temperature and resuspended in 500 μl ice-cold PBS. Lastly, the green (JC-1 monomers) and red (JC-1 aggregates) fluorescence ratio was detected by flow cytometry using a FACSCalibur instrument.

### Quantification of reactive oxygen species and malondialdehyde level

Intracellular reactive oxygen species (ROS) levels were evaluated using dihydroethidium (DHE, YEASEN, 50102ES02), which is one of the most commonly used fluorescence detection probes for superoxide anion and is effective for the detection of ROS, according to the manufacturer’s instructions. In brief, the cells were cultured in 96-well plates and incubated with 10 μM DHE at 37 °C for 30 min. After incubation, the cells were washed 3 times with PBS at room temperature. Intracellular production of ROS in resuspended cells was detected by flow cytometry (BD Biosciences). Malondialdehyde (MDA) is one of the products of lipid peroxidation; it is a secondary product of ROS-induced damage and the ongoing levels of ROS were detected by the levels of MDA. MDA level was evaluated using a cellular MDA detection assay kit (Nanjing Jiancheng Bioengineering Institute, A003–4) using lysed cells. The final density at 532 nm was determined using a microplate reader (Bio-Rad).

### Quantification of superoxide dismutase activity

Cells at a density of 1.0 × 10^6^ cells/well were seeded in six-well plates for 24 h. After that, the cells were harvested, washed twice, and lysed by sonication on ice. After assessment with a superoxide dismutase (SOD) detection assay kit (Nanjing Jiancheng Bioengineering Institute, A001–1), the final density at 550 nm was determined using a microplate reader (Bio-Rad).

### Quantification of lactate dehydrogenase release

Lactate dehydrogenase (LDH) is present in the cytoplasm of normal cells. When the cell membrane is damaged, LDH is released extracellularly and can be detected in the cell culture supernatant. LDH activity can determine the degree of cell damage. LDH level in the cell supernatant was evaluated using an assay kit (Nanjing Jiancheng Bioengineering Institute, A020–2). The final density was determined using a microplate reader (Bio-Rad) at 450 nm.

### Xenograft animal model

Male BALB/c nu/nu nude mice (4 weeks old) were purchased from the Shanghai Laboratory Animal Center and were maintained in a clean environment for 1 week before using them for in vivo experiments. SUDHL-4 cells stably expressing RasGRP4 shRNA-2 or scramble shRNA were resuspended in serum-free medium and mixed with Matrigel basement membrane matrix (BD Biosciences) at a 1:1 ratio (final volume 200 μl), then injected subcutaneously into the right flank of the mice (4 mice/group). Tumor dimensions were measured every 2 days by digital caliper, and the tumor volume (V) was calculated according to the following formula: V = (maximal length × maximal width^2^)/2. One month after implantation, all mice were sacrificed and the xenograft tumors were excised and then weighed. The differences in tumor measurements were assessed between the two groups. Morphological evaluation of tumor tissues was performed using hematoxylin-eosin staining, and the remaining tumor tissues were homogenized for preparing the protein samples to undergo WB analysis. The Shanghai Jiao Tong University School of Medicine Animal Care and Use Committee approved the animal research.

### Collection of clinical data

Clinical data were collected and analyzed for 23 patients with DLBCL. Information regarding patient gender, age, international prognostic index (IPI) or age-adjusted IPI (aaIPI) [19], clinical stage, number of extranodal involved organs, LDH values, largest lesion diameter, the maximum standardized uptake value (SUV_max_), values from positron emission tomography-computed tomography (PET-CT) examination, and the AOD were recorded for each patient. Patients with an IPI score of 0–2 or aaIPI score of 0–1 were considered as part of the low-risk group, and patients with IPI score of 3–5 or aaIPI score of 2–3 were considered as part of the high-risk group. Patients were separated into 2 groups based on a largest lesion diameter of < 10 cm and a largest lesion diameter of ≥10 cm.

### Statistics

Data are expressed as the mean ± standard deviation. Statistical significance between control and treatment groups or between different clinical groups were analyzed using Fisher’s exact test or Student’s *t* test and Kruskal-Wallis test with SAS 9.2 software. Correlation between SUV_max_ and the AOD in DLBCL patients was evaluated by Pearson’s correlation test. *P* < 0.05 was considered statistically significant.

## Results

### Expression of RasGRP4 is the highest among RasGRP family members in lymphoma

**Fig. 1:**
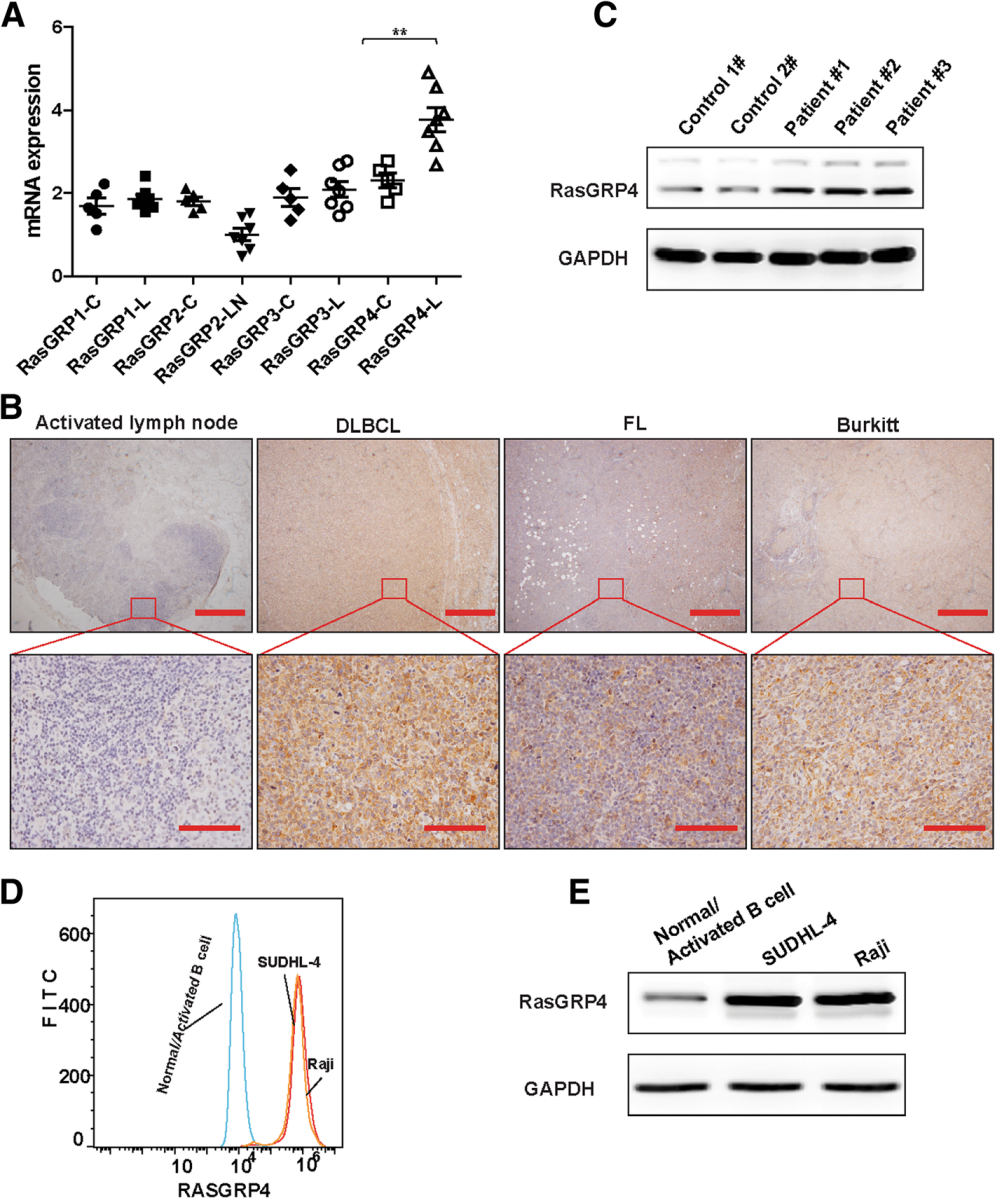
Expression of RasGRP4 in lymphoma and normal tissues. (**a**) RasGRP4 was the most abundantly expressed protein among the RasGRP family members in lymphoma. (**b**) Representative images of the expression of RasGRP4 in DLBCL, FL, and Burkitt lymphoma, which was higher than in reactive lymph nodes. DLBCL showed the highest expression level. Scale bars: 1000 μm (above), 125 μm (below). (**c**) Representative WB images showing that the expression of RasGRP4 protein was significantly higher in DLBCL patients than in controls. (**d** and **e**) Representative images showing that RasGRP4 expression was higher in SUDHL-4 and Raji cell lines compared with normal/activated B cells, as determined by flow cytometry and WB. N indicates the number of independent experiments. **P < 0.01

The mRNA expression of the 4 RasGRP members was evaluated to identify the expression and participation of the 4 RasGRP family members in lymphoma. qRT-PCR was performed using 5 normal or inflammatory reactive lymph node tissues and 7 DLBCL tissues for comparison. The expression levels of RasGRP4 were significantly elevated in the DLCBCL tissues (*P* = 0.003), while RasGRP4 showed the highest expression among the RasGRP family members (Fig. 1a, c: control group, L: lymphoma tissue).

### Increased expression of RasGRP4 in patients with multiple types of B cell lymphoma

To investigate the role of RasGRP4 in the pathogenesis of DLBCL, we immunohistochemically examined RasGRP4 expression in the paraffin-section samples of patients with primary DLBCL. The results showed that the RasGRP4 expression was much higher in DLBCL tissues than in benign tissues of all patients. To further evaluate the expression of RasGRP4 in other types of B cell lymphoma, we examined 5 cases each of follicular lymphoma (FL) and Burkitt lymphoma. Interestingly, the expression of RasGRP4 in FL and Burkitt lymphoma was higher than in reactive lymph nodes (Fig. 1b). However, the expression level of RasGRP4 in FL and Burkitt lymphoma was noticeably lower when compared with DLBCL. Therefore, we selected the DLBCL cell line SUDHL-4 for further study. We collected fresh reactive lymph nodes (lymph nodes were obtained from patients with pathologically confirmed inflammation who underwent surgery) as control tissues and collected fresh DLBCL tissues to examine RasGRP4 expression by WB. The WB results were consistent with IHC results, showing significantly higher RasGRP4 expression in DLBCL tissues than control tissues (Fig. 1c).

### RasGRP4 expression is elevated in lymphoma cell lines

RasGRP4 expression was analyzed in normal or activated B cells sorted from benign lymph nodes in SUDHL-4 lymphoma cells and in Raji cells by flow cytometry and WB. The results showed that RasGRP4 was highly expressed in SUDHL-4 and Raji cell lines (Fig. 1d, e).

### No somatic mutations in RasGRP4 gene

RasGRP4 nucleic acids were extracted from 4 DLBCL patients and 10 monoclones of each patient were selected for sequencing. The result showed that there isn’t any somatic mutation in these patients (Additional file 4).

### Downregulation of RasGRP4 decreases DLBCL cell proliferation

**Fig. 2:**
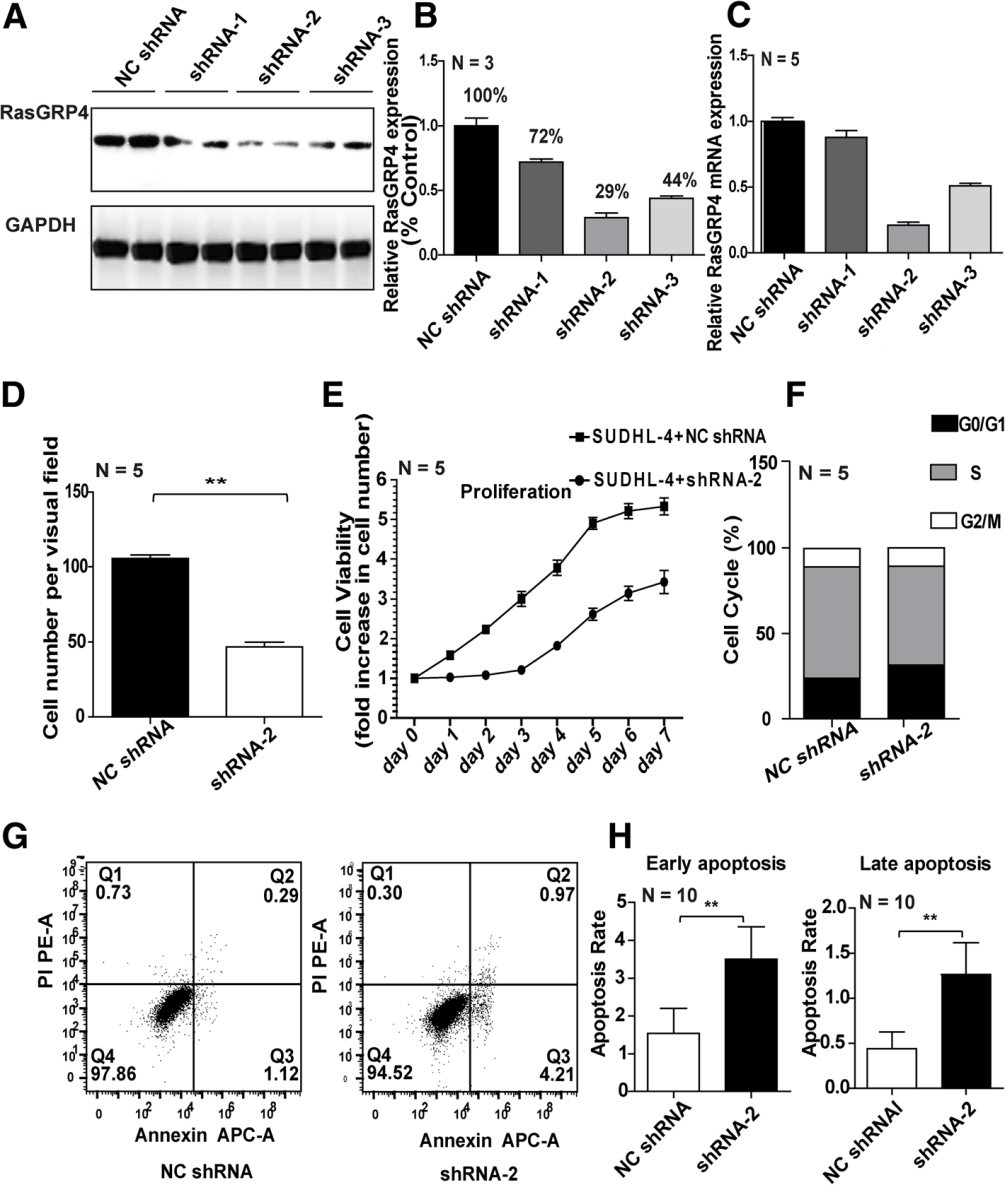
Effective knockdown of RasGRP4 by lentiviral shRNA plasmids and result in decreased DLBCL cell proliferation. (**a**) Representative images of WB analysis, which show the different efficiencies of the three shRNAs in decreasing RasGRP4 expression in DLBCL. (**b**) Quantification of the densitometric assessment of protein bands (in a) using ImageJ software. The expression levels of RasGRP4 after transfection with shRNA-1, − 2, and − 3 were 72, 29, and 44%, respectively. (**c**) RasGRP4 mRNA levels were evaluated by qRT-PCR. N indicates the number of independent experiments. (**d**) Quantification of the cell number in the two groups (in A). The number of DLBCL cells was significantly lower in the RasGRP4-knockdown group compared with control. (**e**) Growth of RasGRP4-knockdown SUDHL-4 cells was significantly slower than the controls. (**f**) RasGRP4 knockdown disrupted the SUDHL-4 cell cycle. (**g**) Representative histograms of the apoptosis rate measured by flow cytometry in the RasGRP4-knockdown and control groups. (**h**) Quantification of apoptosis rate (in **g**), which showed that the proportion of cells in early and late apoptosis was significantly increased by RasGRP4 knockdown. N indicates the number of independent experiments. **P < 0.01

Lentiviral plasmids containing RasGRP4 shRNA and GFP were used to determine the function of RasGRP4 in DLBCL. Cells infected with RasGRP4 shRNA expressed GFP and showed no significant differences in the transfection efficiency between control group and shRNA groups after 72 h of transfection (Additional file 1: Figure S1 A). Based on WB analysis and densitometric evaluation of protein bands using ImageJ software, the RasGRP4-targeting shRNA-2 was considered to be the most efficient shRNA for decreasing RasGRP4 protein expression levels in DLBCL cells (Fig. 2a, b) as well as the mRNA levels (Fig. 2c). Light microscopy was performed to evaluate the proliferation of DLBCL cells and showed significant reduction after knockdown of RasGRP4 by shRNA-2 (Additional file 1: Figure S1 B). The average cell number in the control group was significantly higher after 48 h of infection than the RasGRP4-knockdown group (shRNA group: 46.75 ± 3.172 vs. control group: 105.5 ± 2.398; *P* < 0.0001; Fig. 2d). Proliferation assay of SUDHL-4 cells also showed significant proliferation following RasGRP4 knockdown (Fig. 2e).

### Knockdown of RasGRP4 decreases division and promotes apoptosis in DLBCL cells

**Fig. 3:**
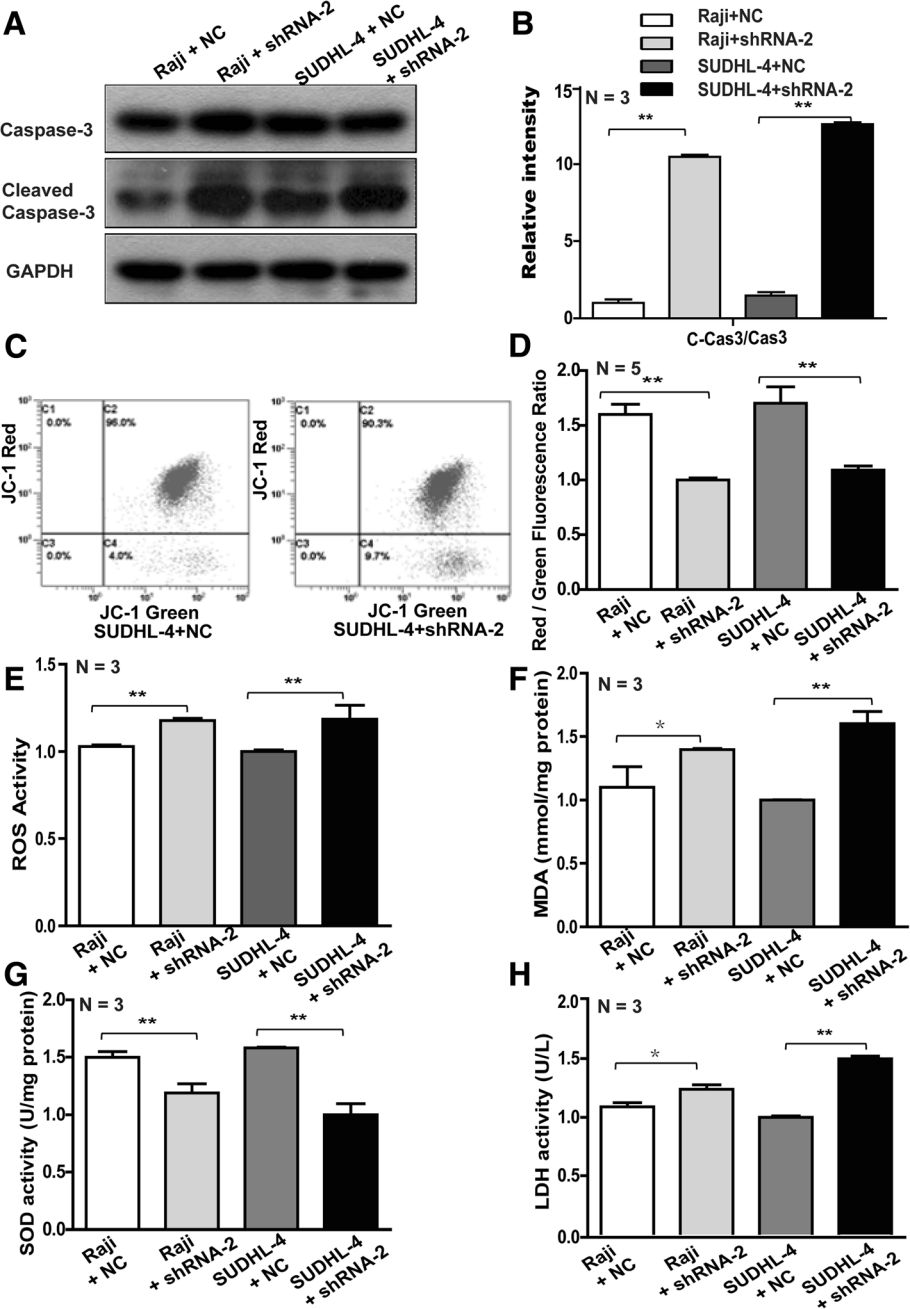
Knockdown of RasGRP4 led to injured mitochondria and upregulated caspase-3 activity and oxidative stress in DLBCL. (**a**) Representative WB images of the cleaved caspase-3 expression in the NC and shRNA groups. (**b**) Quantification of the ratio of cleaved caspase-3/procaspase-3 (in a), which showed that the ratio was significantly higher in RasGRP4-knockdown groups than NC groups after analysis by ImageJ. (**c**) Representative histograms of mitochondrial membrane potential measured by flow cytometry with JC-1 probe. (**d**) Quantification of the red/green fluorescence ratio (in **c**), which was significantly lower when RasGRP4 was downregulated. The levels of oxidative stress markers ROS (**e**), MDA (**f**), and SOD (**g**) were determined using commercial assay kits. RasGRP4 knockdown significantly increased the oxidative stress in lymphoma cells. (**h**) RasGRP4 knockdown resulted in lymphoma cell injury. Intracellular LDH content was significantly higher in the knockdown groups than in the NC groups. N indicates the number of independent experiments. **P < 0.01, *P < 0.05

Cell cycle and apoptosis analysis in SUDHL-4 cells was performed to explore the underlying mechanisms of reduced proliferation of DLBCL cells mediated by RasGRP4 knockdown. In SUDHL-4 cells with RasGRP4 knockdown, the percentage of cells in the G0/G1 phase was increased relative to the control cells (shRNA group: 31.526% ± 3.42% vs. control group: 23.958% ± 1.86%; *P* = 0.0025), and the percentages of cells in S and G2 phases were decreased (shRNA group: 68.472% ± 3.42% vs. control group: 76.042% ± 1.86%; *P* = 0.0025; Fig. 2f). Both early and late apoptosis were significantly increased in the RasGRP4-knockdown groups compared to the NC groups (early apoptosis: NC 1.54 ± 0.66 vs. shRNA 3.5 ± 0.84, *P* < 0.001; late apoptosis: NC 0.44 ± 0.19 vs. shRNA 1.26 ± 0.35, *P* < 0.001; Figs. 2g, 3h). At the same time, the protein levels of cleaved caspase-3 in SUDHL-4 and Raji cells with RasGRP4 knockdown showed significant upregulation compared with the NC shRNA groups (Fig. 3a). Based on the densitometric evaluation with ImageJ software and statistical analysis, the average cleaved caspase-3 expression level in the RasGRP4-knockdown groups was significantly higher than that of the control groups (SUDHL-4 and Raji: *P* < 0.0001; Fig. 3b). In addition, the apoptotic mechanism was further explored by applying JC-1 probe to detect the mitochondrial membrane potential. In normal mitochondria, JC-1 was exists as a polymer in the mitochondrial matrix and emits intense red fluorescence (Ex = 585 nm, Em = 590 nm). In unhealthy mitochondria resulting from membrane potential decrease or loss, JC-1 exists as a monomer in the cytosol, producing green fluorescence (Ex = 514 nm, Em = 529 nm). Thus, the change in color of JC-1 can directly reflect the changes in the mitochondrial membrane potential. The results in Fig. 3c and d show that the downregulation of RasGRP4 significantly reduced the ratio of red fluorescence (high mitochondrial membrane potential; SUDHL-4 and Raji: *P* < 0.0001), indicating that the mitochondria were injured.

### Knockdown of RasGRP4 increases the oxidative stress levels in lymphoma cells

Next, we investigated the oxidative stress levels in SUDHL-4 and Raji cells to explore the mechanism by which downregulation of RasGRP4 leads to increased damage of lymphoma cells. The results showed that the ROS activity was significantly increased (Fig. 3e, SUDHL-4: *P* = 0.0002; Raji: *P* = 0.0006) after the expression of RasGRP4 was decreased by shRNA. Furthermore, MDA levels showed a similar change (Fig. 3f, SUDHL-4: *P* = 0.0022; Raji: *P* = 0.011). In contrast, the activity of SOD was significantly reduced when downregulating RasGRP4 in lymphoma cells (Fig. 3g, SUDHL-4: *P* = 0.0005; Raji: *P* = 0.0015). LDH is considered one of the major representative indicators of cell injury, and so we measured the intracellular LDH content. Figure 3h shows that the downregulation of RasGRP4 markedly increased the LDH leakage from lymphoma cells (SUDHL-4: *P* = 0.0007; Raji: *P* = 0.047).

### MAPK-associated signaling pathway activation participates in DLBCL

**Fig. 4:**
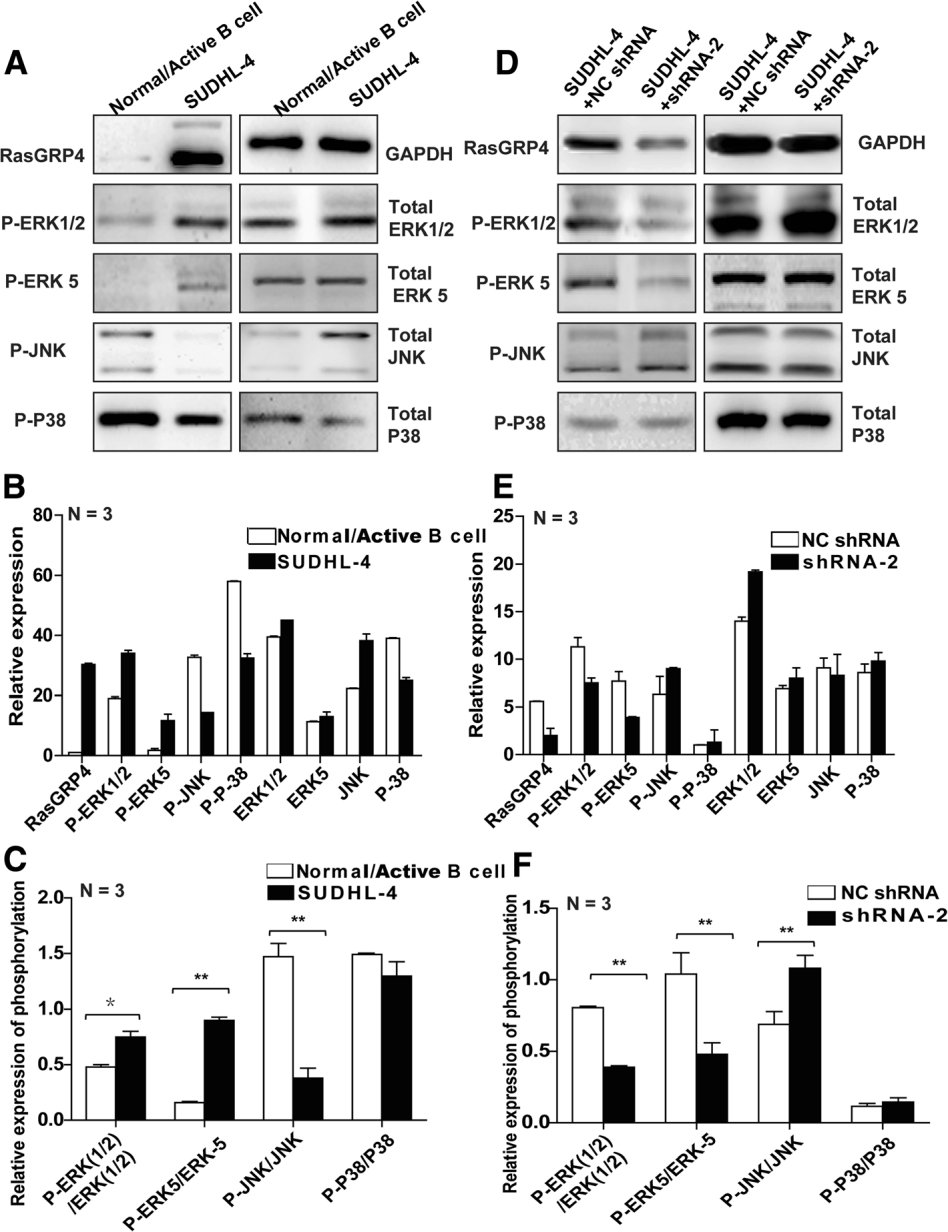
MAPK-associated signaling pathways downstream of RasGRP4 are involved in DLBCL. (**a**) Representative WB images showing the differences in expression and phosphorylation levels of the downstream signaling proteins between SUDHL-4 cells and normal/activated B cells. (**b** and **c**) Quantification of the levels (in **a**) and the phosphorylation ratio (in **a**). Compared with normal/activated B cells, the ratios of P-ERK1/2:ERK1/2 and P-ERK5:ERK5 were increased in DLBCL cells, while the ratio of P-JNK:JNK was decreased, and no significant change in the P-P38:P38 ratio was detected. (**d**) Representative WB images showing the differences in expression and phosphorylation levels of the downstream signaling proteins in RasGRP4-knockdown SUDHL-4 cells and the control group. (**e** and **f**) Quantification of the levels (in **d**) and the phosphorylation ratio (in **d**). Knockdown of RasGRP4 decreased the ERK expression and increased the JNK expression in SUDHL-4 cells; densitometry was analyzed using ImageJ software. N indicates the number of independent experiments. **P < 0.01, *P < 0.05

Previous reports suggested that the MAPK pathway was constitutively activated and played a critical role as a downstream signaling pathway of RasGRP family members in various neoplasms [20, 21]. To investigate whether the MAPK pathway is involved in DLBCL, we evaluated phosphorylation of MAPK signaling proteins in SUDHL-4 cells and normal or activated B cells by WB and compared the band intensity by densitometry. The phosphorylated (P)-ERK1/2:ERK1/2 and P-ERK5:ERK5 ratios were increased in the DLBCL cell line, while the P-JNK:JNK ratio was decreased, and no significant difference in the P-P38:P38 ratio was observed (Fig. 4a–c).

### Knockdown of RasGRP4 decreases ERK expression and increases expression of JNK in SUDHL-4 cells

To further verify the activation of the MAPK signaling pathway in DLBCL that is involved in mediating the effects of RasGRP4, phosphorylation of various MAPKs was evaluated in DLBCL cells by knockdown of RasGRP4 and compared with the NC group. The results of MAPK phosphorylation showed a similar trend to the phosphorylation observed in DLBCL and normal/activated B cells, indicating a significant decrease in the P-ERK:ERK ratio and increase in the P-JNK:JNK ratio in RasGRP4-knockdown SUDHL-4 cells (Fig. 4d–f).

### Knockdown of RasGRP4 inhibits DLBCL growth in vivo

**Fig. 5:**
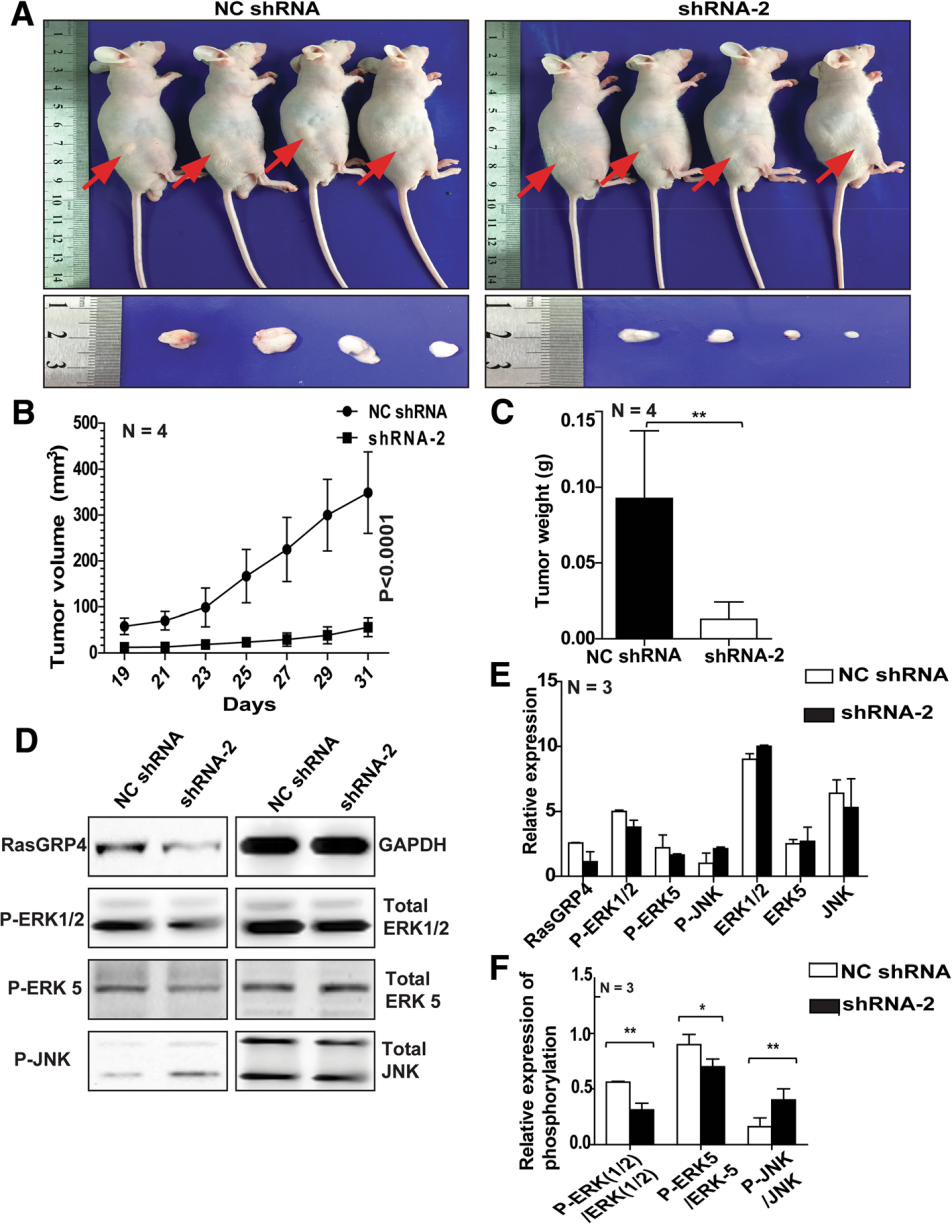
Knockdown of RasGRP4 inhibits DLBCL growth in vivo. (**a**) Knockdown of RasGRP4 led to a profound suppression of tumor growth in the shRNA-2 group. (**b** and **c**) Tumor volume and weight were significantly reduced in the shRNA-2 group. (**d**) Representative images of WB analysis of the MAPK signaling pathway in tumor tissues isolated from mice bearing RasGRP4-knockdown tumors and control tumors. (**e**) Quantification of ERK, JNK, and their phosphorylation (in **d**). (**f**) Quantification of the phosphorylation ratio for ERK and JNK (in d). Decrease in the P-ERK:ERK ratio and increase in the P-JNK:JNK ratio was observed in tumor tissues with RasGRP4-knockdown cells. Densitometry was assessed using ImageJ software. N indicates the number of independent experiments. **P < 0.01, *P < 0.05

To identify the effects of RasGRP4 on DLBCL tumor growth in vivo, we engrafted SUDHL-4 cells with RasGRP4-knockdown or NC cells into nude mice. Knockdown of RasGRP4 led to a profound suppression of tumor growth in vivo (Fig. 5a). Tumor volume and weight were significantly reduced by RasGRP4 knockdown. When the tumor size and weight of the experimental group and the control group were evaluated on day 31, the difference showed statistical significance (*P* < 0.0001 and *P* = 0.0001, respectively; Fig. 5b, c). Furthermore, WB analysis showed a reduced P-ERK:ERK ratio and an increased P-JNK:JNK ratio in tumor tissues from mice bearing RasGRP4-knockdown tumors (Fig. 5d, e).

### High RasGRP4 expression is associated with tumor size and poor prognosis in DLBCL

**Fig. 6:**
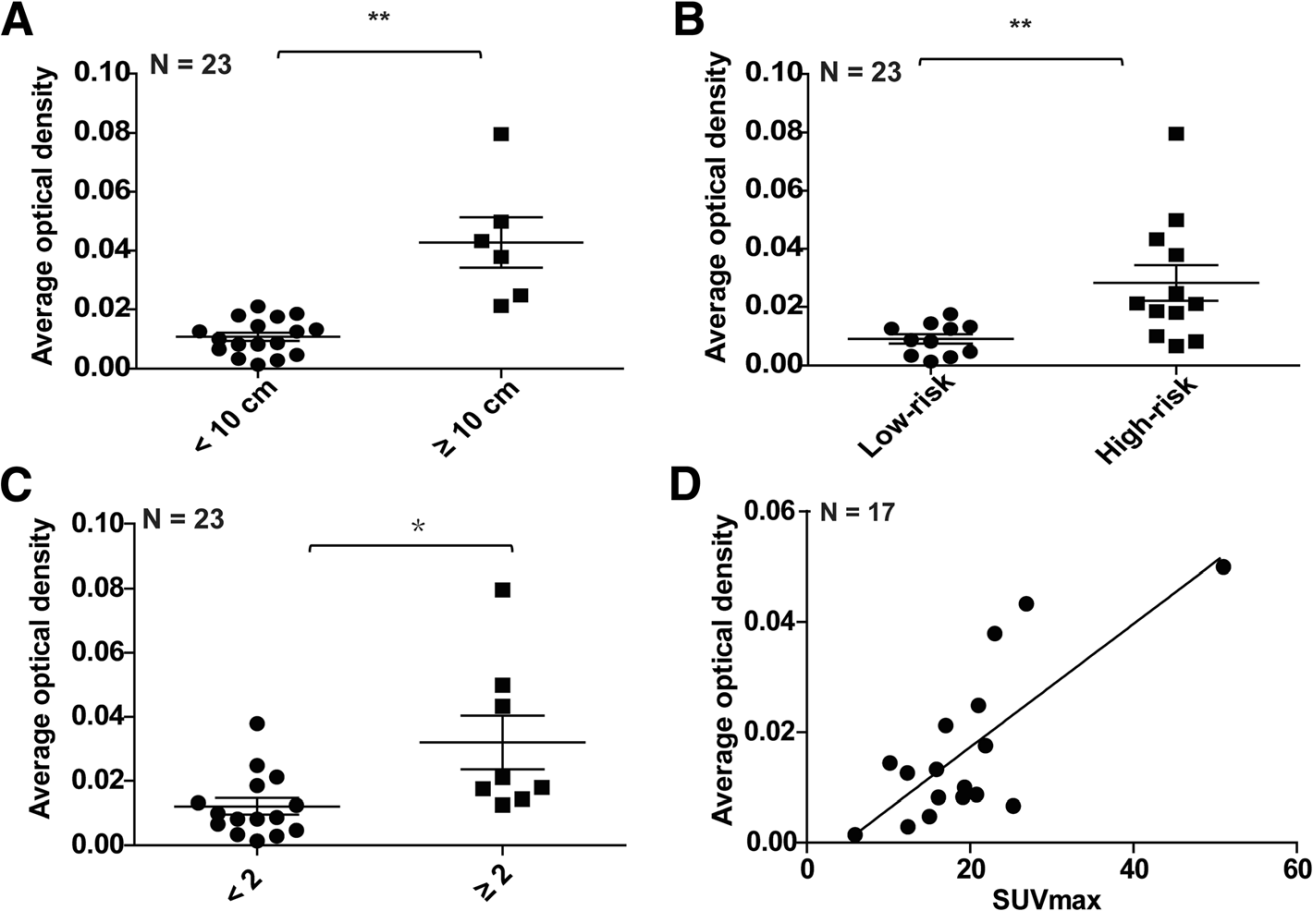
High expression of RasGRP4 was associated with tumor size and poor prognosis in DLBCL patients. (**a**) The expression level of RasGRP4 was significantly higher in DLBCL patients with the largest lesion diameter of ≥10 cm (*P* = 0.0004). (**b**) RasGRP4 expression was significantly higher in patients with a high-risk IPI (or aaIPI) score (*P* = 0.0042). (**c**) RasGRP4 expression was significantly different when the number of extranodal organs involved was greater than 2 (*P* = 0.0108). (**d**) The expression of RasGRP4 was positively correlated with SUV_max_ value in patients with DLBCL (*P* < 0.001). N indicates the number of independent experiments. **P < 0.01, *P < 0.05

Comprehensive clinical information was collected from 23 patients with DLBCL to explore the relationship between RasGRP4 expression and clinical characteristics. Clinical characteristics, including sex, age, IPI or aaIPI, clinical stage, number of involved extranodal organs, LDH value, largest lesion diameter, SUV_max_ value, and RasGRP4 expression level (reflecting by the AOD of each patient) were recorded and statistically analyzed. The results showed that the RasGRP4 expression was significantly higher in DLBCL patients with a largest lesion diameter of ≥10 cm compared to those with the largest lesion diameter of < 10 cm (*P* = 0.0004, Fig. 6a, staining data in Additional file 2: Figure S2). The expression of RasGRP4 was significantly higher in patients with high-risk IPI (or aaIPI) score (*P* = 0.0042; Fig. 6b, staining data in Additional file 3: Figure S3). To further explore the related influential factors, we analyzed RasGRP4 expression differences by IPI, including age, stage, extranodal involvement, and LDH level. The results revealed that the RasGRP4 expression was significantly different when the extranodal involvement was independently considered (*P* = 0.0108; Fig. 6c). We also found that the expression of RasGRP4 was positively correlated with SUV_max_ value in patients with DLBCL (*P* < 0.001; Fig. 6d). The detailed clinical data and RasGRP4 staining data for each patient are presented in Table 2.

**Table 2:** Baseline characteristics of DLBCL patients

Patient	Gender	Age	IPI/aa IPI	Stage	Extranodal Involved Organs	LDH	Maximum Diameter (cm)	SUV_max_ Value	AOD (Rasgrp4 expression)
1	F	72	4	IVB	1	(−)	3	16.1	0.00819
2	M	66	3	IIIA	1	(+)	6	21	0.02483
3	F	76	3	IV	1	(+)	7.8	23	0.03785
4	M	22	2	IVB	3	(+)	14.8	26.9	0.04329
5	F	61	3	IIIA	0	(+)	10.5	17	0.02118
6	M	59	3	IV	2	(+)	7.5	/	0.02108
7	F	70	2	IVA	1	(+)	3	5.9	0.00141
8	F	69	4	IIIB	0	/	4.5	19.3	0.00998
9	M	64	1	IVA	2	(+)	6.6	21.9	0.01752
10	M	37	2	IV	1	/	4	25.3	0.00663
11	F	46	2	IV	2	(−)	19	/	0.0796
12	F	70	2	IIIB	0	(−)	4	/	0.00336
13	F	68	1	IIA	0	(+)	3.3	12.4	0.00286
14	F	54	0	IVA	2	(−)	5	12.3	0.01258
15	F	80	3	IIB	0	(+)	8	/	0.01853
16	M	58	1	IVA	1	(−)	4.5	20.8	0.00869
17	F	59	0	IV	2	(+)	5	10.2	0.01436
18	F	30	1	IIA	0	(−)	6.3	/	0.01246
19	M	74	2	III	0	/	2.8	15	0.0047
20	F	55	2	IV	2	(+)	4	/	0.01799
21	F	63	3	IVA	2	(−)	23	51	0.0499
22	M	43	0	IIA	0	/	3.7	19.1	0.00823
23	F	30	1	IIA	0	(−)	5.2	15.9	0.01324

*F* female, *M* male, *IPI* International Prognostic Index, *aaIPI* Age-adjusted International Proghostic Index, *(+)*: higher than normal level, *(−)*: normal level, *AOD* Average optical density

## Discussion

As DLBCL showed the highest morbidity of all B cell lymphoma subtypes in Asia, it makes our research more meaningful. We found marked elevation of RasGRP4 expression, which indicating that it may have potential clinical significance in DLBCL.

We not only demonstrated the antitumor activity of RasGRP4 both in vitro and in vivo*,* but also explored the possible upstream and downstream mechanisms of RasGRP4 in DLBCL. There are no somatic mutations in our sequencing result from DLBCL patients, which indicates that there must be some upstream regulation that increases the expression of RasGRP4. In order to find out the mechanism of higher RasGRP4 expression in DLBCL, we examined the expression levels and phosphorylation difference of upstream protein (PLC-γ1) and possible promoter (NF-κB p65 and c-jun) of RASGRP4. Our data showed that there is no obvious difference of phospho-PLC-γ1 and phospho-c-jun, while the phosphorylated NF-κB p65: NF-κB p65 ratio was developed in 2 of 4 DLBCL patients (data not shown). However, due to limited clinical samples and individual differences in samples, specific upstream regulatory mechanisms will continue to be explored in future studies.

We found that the MAPK-associated signaling pathways are activated in DLBCL compared with normal or activated B cells. This was evidenced by higher levels of ERK phosphorylation and lower JNK phosphorylation. The opposite phenomenon was observed when RasGRP4 was knocked down in SUDHL-4 cells, demonstrating its role in the MAPK pathway downstream of DLBCL oncogenesis. Moreover, WB analysis of tumor tissues from xenograft studies showed similar alterations in MAPK signaling. The MAPK (ERK/JNK) pathway is commonly activated downstream of RAS, and is constitutively activated during cell proliferation, differentiation, migration, senescence, and apoptosis in various cancers [22, 23]. However, the regulation of RAS is still unclear, and as an upstream mediator of activation of oncogenic pathways, RAS has been largely unexplored as a potential therapeutic target in lymphoma. Additionally, RasGRPs are the upstream molecules that regulate RAS-MAPK activation in other lymphocyte malignancies [20, 21]. Thus, our data along with data from previous reports helps to explain the oncogenic role of RasGRP4 in cell proliferation and tumor growth of DLBCL. This in turn suggested that RasGRP4 plays a crucial role in the formation and progression of DLBCL, acting through downstream ERK and JNK signaling pathways.

To further explore the potential clinical value of RasGRP4 in DLBCL, we compared the associations of RasGRP4 expression with comprehensive clinical information. We found that elevated RasGRP4 expression was associated with bigger lesions, which was measured by CT images. This may suggest an association between RasGRP4 expression and proliferative capacity of lymphoma cells and was consistent with our in vitro observations. IPI is a clinical indicator and an important prognostic factor for patient survival. This was informed by five clinical characteristics: age, WHO performance status, stage, extranodal involvement, and LDH levels. IPI is routinely applied clinically and is often applied in research as a prognostic indicator for DLBCL and other lymphomas [24]. The aaIPI is a similar international universal index and is used to evaluate the comprehensive condition of young patients (age ≤ 60). IPI 0–2 or aaIPI 0–1 is considered low risk, while IPI 3–5 or aaIPI 2–3 is considered high risk. In our study, RasGRP4 expression was significantly higher in the high-risk group, indicating that high RasGRP4 expression was correlated with poor prognosis in DLBCL patients. To further explore the relationship between RasGRP4 expression and specific characteristics of IPI, an independent analysis of component elements of IPI referring to the scoring criteria (age ≤ 60 vs. > 60; stage I/II vs. stage III/IV; the number of extranodal involved organs < 2 vs. ≥ 2; normal LDH level vs. abnormal) was performed. The results showed significant differences in RasGRP4 expression between different extranodal involvement groups, supporting the conclusion that RasGRP4 has prognostic significance. We also recorded the SUV_max_ value of patients using PET-CT scan. PET-CT is the recommended imaging method to be used clinically when evaluating lymphoma patients [25, 26], and the SUV_max_ value showed correlation with prognosis and lymphoma recurrence [27–29]. Our data showed that RasGRP4 expression was positively correlated with the SUV_max_ value, supporting the association with high-risk IPI groups, further demonstrating the prognostic value of RasGRP4.

## Conclusion

Our results demonstrated that the expression of RasGRP4 is elevated in DLBCL tumor tissues, suggesting a novel oncogenic role of RasGRP4 in DLBCL. This finding should be further verified in future studies with larger patient populations. Our novel demonstration that RasGRP4 may play a crucial role in DLBCL growth raises the exciting possibility that RasGRP4 inhibition may be a therapeutic approach in DLBCL.

## Additional files

Additional file 1: **Figure S1.** (**A**) Representative images of SUDHL-4 cells after 72 h of transfection. The cells infected with RasGRP4 shRNA displayed green fluorescence and there were no significant differences in the transfection efficiency between the control and shRNA groups. Scale bars: 500 μm. (**B**) Representative images of SUDHL-4 cells after 48 h of transfection. The number of DLBCL cells in visual field was significantly lower in the RasGRP4-knockdown group compared with control. Scale bars: 500 μm. (TIF 76125 kb)

Additional file 2: **Figure S2.** Detailed data of RasGRP4 staining of each patient in the largest lesion diameter of < 10 cm (**A**) and ≥ 10 cm (**B**) groups. (TIF 87481 kb)

Additional file 3: **Figure S3.** (**A**) Detailed RasGRP4 staining of IHC figures and corresponding AOD scores (measured with Image-Pro plus 6.0 software) of each patient in the low risk IPI score groups. (**B**) Detailed RasGRP4 staining IHC figures and corresponding AOD score of each patient in the high risk IPI score groups. (TIF 87627 kb)

Additional file 4: Sequencing results of RasGRP4 from 4 patients with DLBCL. (DOC 37 kb)

## Data Availability

All data generated or analysed during this study are included in this published article [and its supplementary information files].

## Abbreviations

DLBCL: Diffuse large B cell lymphoma
ERK: Extracellular signal-regulated kinase
JNK: c-Jun N-terminal kinase
MAPK: Mitogen-activated protein kinase
RasGRP4: Ras guanine nucleotide-releasing protein 4
PBS: Phosphate-buffered saline
IHC: Immunohistochemistry
ROS: Reactive oxygen species
MDA: Malondialdehyde
AOD: Average optical density
IPI: International prognostic index
SUVmax: Maximum standardized uptake value
LDH: Lactate dehydrogenase
