# Supplementary material for: The critical role of RasGRP4 in the growth of diffuse large B cell lymphoma
Source: Cell Commun Signal. 2019 Aug 13;17:92. doi: 10.1186/s12964-019-0415-6 (PMC6693169; doi:10.1186/s12964-019-0415-6)
Supplement: Supplementary file 4 — Sequencing results of RasGRP4 from 4 patients with DLBCL. (DOC 37 kb) [file 12964_2019_415_MOESM4_ESM.doc]

RASGPR4

1-2

atgaacagaaaagacagtaagaggaagtcccaccaggaatgcaccggaaaaacaggagggcgaggccggccccgccaagtgcgccgccacaagacatgccccagccctcgggaaatcagcaaggtcatggcttccatgaacctgggcctgctgagtgagggcggctgcagcgaagatgagctgctggagaaatgcatccagtccttcgattcagctggcagcctgtgccacgaggaccacatgctcaacatggtgctggccatgcacagctgggtgctgccgtccgccgacctggctgcccgcctgctgacctcataccagaaggccacaggggacacccaggagctgagacggctgcagatctgtcacctggtcaggtactggctgatgcgacaccctgaggtgatgcaccaggatccccagctagaagaagtcataggtcgtttctgggccaccgtggcccgggagggcaactcagcccagagaagactgggagactcttctgacctcccaggcctgggcaaaaagcgcaaagtgtccttgcttttcgaccacttggagacgggggagctggctcagcacctcacctacctggagttccggtccttccaggctatcacgccccaggacctgcggagctacgttttgcagggctcagtacgaggctgcccggccctggagggctccgtaggtctcagcaacagcgtgtcccgctgggtgcaggtgatggtgctgagccgtcccgggcccctacagcgtgcacaggtgctggacaagttcattcacgtggcacagaggctccaccagctgcagaatttcaacacgctgatggcagtcacagggggcctgtgtcacagtgccatctccagactcaaggactcccatgcccacctgagccctgacagcaccaaggccctcctggagctcactgagctccttgcctcccacaacaactacgcccgctaccgccgcacctgggctggctgcgcgggtttccggctgcctgtactgggcgtgcacctcaaggacctggtgtccctgcatgaggcacagcccgacaggttgcctgacggccgcctgcacctacccaagctgaacaacctctacctgcggctgcaggagctggtggccctccaagggcagcatccaccctgcagcgccaatgaggatctgctgcacctgctcacgctctccctggacctcttctacacggaagacgagatctatgagctttcttatgcccgggagccgcgttgtcccaagagcctgccaccctcccccttcaatgcacctctggtggtggagtgggcccctggtgtgacacccaagccggacagggtcacactgggtcggcatgtggagcagctggtggagtctgtgttcaagaattatgaccctgaaggccgaggaacaatctctcaggaggactttgagcgactctcgggcaattttcccttcgcctgccatgggcttcacccacccccacgccaggggagaggatccttcagcagagaggagctgacagggtacctgctccgggccagcgccatctgctccaagttgggcctggccttcctgcacaccttccatgaggtcaccttccgaaagcctaccttctgcgacagctgcagtggcttcctctggggtgtcaccaagcaaggctaccgctgtcgggagtgcgggctgtgttgccacaaacactgcagagaccaggtgaaggtagaatgtaagaagaggccaggggccaagggcgatgcaggaccccccggagctcctgtcccatccacaccagctccccatgccagctgtggctccgaggaaaatcactcctacacgctatccctggagcctgagactgggtgccagcttcgccatgcctggacccagactgaatccccacacccttcctgggaaacagatacggtcccctgcccggtgatggacccaccatcaactgcatcctccaagctggattcctag

2-3

Atgaacagaaaagacagtaagaggaagtcccaccaggaatgcaccggaaaaacaggagggcgaggccggccccgccaagtgcgccgccacaagacatgccccagccctcgggaaatcagcaaggtcatggcttccatgaacctgggcctgctgagtgagggcggctgcagcgaagatgagctgctggagaaatgcatccagtccttcgattcagctggcagcctgtgccacgaggaccacatgctcaacatggtgctggccatgcacagctgggtgctgccgtccgccgacctggctgcccgcctgctgacctcataccagaaggccacaggggacacccaggagctgagacggctgcagatctgtcacctggtcaggtactggctgatgcgacaccctgaggtgatgcaccaggatccccagctagaagaagtcataggtcgtttctgggccaccgtggcccgggagggcaactcagcccagagaagactgggagactcttctgacctcccaggcctgggcaaaaagcgcaaagtgtccttgcttttcgaccacttggagacgggggagctggctcagcacctcacctacctggagttccggtccttccaggctatcacgccccaggacctgcggagctacgttttgcagggctcagtacgaggctgcccggccctggagggctccgtaggtctcagcaacagcgtgtcccgctgggtgcaggtgatggtgctgagccgtcccgggcccctacagcgtgcacaggtgctggacaagttcattcacgtggcacagaggctccaccagctgcagaatttcaacacgctgatggcagtcacagggggcctgtgtcacagtgccatctccagactcaaggactcccatgcccacctgagccctgacagcaccaaggccctcctggagctcactgagctccttgcctcccacaacaactacgcccgctaccgccgcacctgggctggctgcgcgggtttccggctgcctgtactgggcgtgcacctcaaggacctggtgtccctgcatgaggcacagcccgacaggttgcctgacggccgcctgcacctacccaagctgaacaacctctacctgcggctgcaggagctggtggccctccaagggcagcatccaccctgcagcgccaatgaggatctgctgcacctgctcacgctctccctggacctcttctacacggaagacgagatctatgagctttcttatgcccgggagccgcgttgtcccaagagcctgccaccctcccccttcaatgcacctctggtggtggagtgggcccctggtgtgacacccaagccggacagggtcacactgggtcggcatgtggagcagctggtggagtctgtgttcaagaattatgaccctgaaggccgaggaacaatctctcaggaggactttgagcgactctcgggcaattttcccttcgcctgccatgggcttcacccacccccacgccaggggagaggatccttcagcagagaggagctgacagggtacctgctccgggccagcgccatctgctccaagttgggcctggccttcctgcacaccttccatgaggtcaccttccgaaagcctaccttctgcgacagctgcagtggcttcctctggggtgtcaccaagcaaggctaccgctgtcgggagtgcgggctgtgttgccacaaacactgcagagaccaggtgaaggtagaatgtaagaagaggccaggggccaagggcgatgcaggaccccccggagctcctgtcccatccacaccagctccccatgccagctgtggctccgaggaaaatcactcctacacgctatccctggagcctgagactgggtgccagcttcgccatgcctggacccagactgaatccccacacccttcctgggaaacagatacggtcccctgcccggtgatggacccaccatcaactgcatcctccaagctggattcctag

2-4

atgaacagaaaagacagtaagaggaagtcccaccaggaatgcaccggaaaaacaggagggcgaggccggccccgccaagtgcgccgccacaagacatgccccagccctcgggaaatcagcaaggtcatggcttccatgaacctgggcctgctgagtgagggcggctgcagcgaagatgagctgctggagaaatgcatccagtccttcgattcagctggcagcctgtgccacgaggaccacatgctcaacatggtgctggccatgcacagctgggtgctgccgtccgccgacctggctgcccgcctgctgacctcataccagaaggccacaggggacacccaggagctgagacggctgcagatctgtcacctggtcaggtactggctgatgcgacaccctgaggtgatgcaccaggatccccagctagaagaagtcataggtcgtttctgggccaccgtggcccgggagggcaactcagcccagagaagactgggagactcttctgacctcccaggcctgggcaaaaagcgcaaagtgtccttgcttttcgaccacttggagacgggggagctggctcagcacctcacctacctggagttccggtccttccaggctatcacgccccaggacctgcggagctacgttttgcagggctcagtacgaggctgcccggccctggagggctccgtaggtctcagcaacagcgtgtcccgctgggtgcaggtgatggtgctgagccgtcccgggcccctacagcgtgcacaggtgctggacaagttcattcacgtggcacagaggctccaccagctgcagaatttcaacacgctgatggcagtcacagggggcctgtgtcacagtgccatctccagactcaaggactcccatgcccacctgagccctgacagcaccaaggccctcctggagctcactgagctccttgcctcccacaacaactacgcccgctaccgccgcacctgggctggctgcgcgggtttccggctgcctgtactgggcgtgcacctcaaggacctggtgtccctgcatgaggcacagcccgacaggttgcctgacggccgcctgcacctacccaagctgaacaacctctacctgcggctgcaggagctggtggccctccaagggcagcatccaccctgcagcgccaatgaggatctgctgcacctgctcacgctctccctggacctcttctacacggaagacgagatctatgagctttcttatgcccgggagccgcgttgtcccaagagcctgccaccctcccccttcaatgcacctctggtggtggagtgggcccctggtgtgacacccaagccggacagggtcacactgggtcggcatgtggagcagctggtggagtctgtgttcaagaattatgaccctgaaggccgaggaacaatctctcaggaggactttgagcgactctcgggcaattttcccttcgcctgccatgggcttcacccacccccacgccaggggagaggatccttcagcagagaggagctgacagggtacctgctccgggccagcgccatctgctccaagttgggcctggccttcctgcacaccttccatgaggtcaccttccgaaagcctaccttctgcgacagctgcagtggcttcctctggggtgtcaccaagcaaggctaccgctgtcgggagtgcgggctgtgttgccacaaacactgcagagaccaggtgaaggtagaatgtaagaagaggccaggggccaagggcgatgcaggaccccccggagctcctgtcccatccacaccagctccccatgccagctgtggctccgaggaaaatcactcctacacgctatccctggagcctgagactgggtgccagcttcgccatgcctggacccagactgaatccccacacccttcctgggaaacagatacggtcccctgcccggtgatggacccaccatcaactgcatcctccaagctggattcctag

3-1

Atgaacagaaaagacagtaagaggaagtcccaccaggaatgcaccggaaaaacaggagggcgaggccggccccgccaagtgcgccgccacaagacatgccccagccctcgggaaatcagcaaggtcatggcttccatgaacctgggcctgctgagtgagggcggctgcagcgaagatgagctgctggagaaatgcatccagtccttcgattcagctggcagcctgtgccacgaggaccacatgctcaacatggtgctggccatgcacagctgggtgctgccgtccgccgacctggctgcccgcctgctgacctcataccagaaggccacaggggacacccaggagctgagacggctgcagatctgtcacctggtcaggtactggctgatgcgacaccctgaggtgatgcaccaggatccccagctagaagaagtcataggtcgtttctgggccaccgtggcccgggagggcaactcagcccagagaagactgggagactcttctgacctcccaggcctgggcaaaaagcgcaaagtgtccttgcttttcgaccacttggagacgggggagctggctcagcacctcacctacctggagttccggtccttccaggctatcacgccccaggacctgcggagctacgttttgcagggctcagtacgaggctgcccggccctggagggctccgtaggtctcagcaacagcgtgtcccgctgggtgcaggtgatggtgctgagccgtcccgggcccctacagcgtgcacaggtgctggacaagttcattcacgtggcacagaggctccaccagctgcagaatttcaacacgctgatggcagtcacagggggcctgtgtcacagtgccatctccagactcaaggactcccatgcccacctgagccctgacagcaccaaggccctcctggagctcactgagctccttgcctcccacaacaactacgcccgctaccgccgcacctgggctggctgcgcgggtttccggctgcctgtactgggcgtgcacctcaaggacctggtgtccctgcatgaggcacagcccgacaggttgcctgacggccgcctgcacctacccaagctgaacaacctctacctgcggctgcaggagctggtggccctccaagggcagcatccaccctgcagcgccaatgaggatctgctgcacctgctcacgctctccctggacctcttctacacggaagacgagatctatgagctttcttatgcccgggagccgcgttgtcccaagagcctgccaccctcccccttcaatgcacctctggtggtggagtgggcccctggtgtgacacccaagccggacagggtcacactgggtcggcatgtggagcagctggtggagtctgtgttcaagaattatgaccctgaaggccgaggaacaatctctcaggaggactttgagcgactctcgggcaattttcccttcgcctgccatgggcttcacccacccccacgccaggggagaggatccttcagcagagaggagctgacagggtacctgctccgggccagcgccatctgctccaagttgggcctggccttcctgcacaccttccatgaggtcaccttccgaaagcctaccttctgcgacagctgcagtggcttcctctggggtgtcaccaagcaaggctaccgctgtcgggagtgcgggctgtgttgccacaaacactgcagagaccaggtgaaggtagaatgtaagaagaggccaggggccaagggcgatgcaggaccccccggagctcctgtcccatccacaccagctccccatgccagctgtggctccgaggaaaatcactcctacacgctatccctggagcctgagactgggtgccagcttcgccatgcctggacccagactgaatccccacacccttcctgggaaacagatacggtcccctgcccggtgatggacccaccatcaactgcatcctccaagctggattcctag

3-2

Atgaacagaaaagacagtaagaggaagtcccaccaggaatgcaccggaaaaacaggagggcgaggccggccccgccaagtgcgccgccacaagacatgccccagccctcgggaaatcagcaaggtcatggcttccatgaacctgggcctgctgagtgagggcggctgcagcgaagatgagctgctggagaaatgcatccagtccttcgattcagctggcagcctgtgccacgaggaccacatgctcaacatggtgctggccatgcacagctgggtgctgccgtccgccgacctggctgcccgcctgctgacctcataccagaaggccacaggggacacccaggagctgagacggctgcagatctgtcacctggtcaggtactggctgatgcgacaccctgaggtgatgcaccaggatccccagctagaagaagtcataggtcgtttctgggccaccgtggcccgggagggcaactcagcccagagaagactgggagactcttctgacctcccaggcctgggcaaaaagcgcaaagtgtccttgcttttcgaccacttggagacgggggagctggctcagcacctcacctacctggagttccggtccttccaggctatcacgccccaggacctgcggagctacgttttgcagggctcagtacgaggctgcccggccctggagggctccgtaggtctcagcaacagcgtgtcccgctgggtgcaggtgatggtgctgagccgtcccgggcccctacagcgtgcacaggtgctggacaagttcattcacgtggcacagaggctccaccagctgcagaatttcaacacgctgatggcagtcacagggggcctgtgtcacagtgccatctccagactcaaggactcccatgcccacctgagccctgacagcaccaaggccctcctggagctcactgagctccttgcctcccacaacaactacgcccgctaccgccgcacctgggctggctgcgcgggtttccggctgcctgtactgggcgtgcacctcaaggacctggtgtccctgcatgaggcacagcccgacaggttgcctgacggccgcctgcacctacccaagctgaacaacctctacctgcggctgcaggagctggtggccctccaagggcagcatccaccctgcagcgccaatgaggatctgctgcacctgctcacgctctccctggacctcttctacacggaagacgagatctatgagctttcttatgcccgggagccgcgttgtcccaagagcctgccaccctcccccttcaatgcacctctggtggtggagtgggcccctggtgtgacacccaagccggacagggtcacactgggtcggcatgtggagcagctggtggagtctgtgttcaagaattatgaccctgaaggccgaggaacaatctctcaggaggactttgagcgactctcgggcaattttcccttcgcctgccatgggcttcacccacccccacgccaggggagaggatccttcagcagagaggagctgacagggtacctgctccgggccagcgccatctgctccaagttgggcctggccttcctgcacaccttccatgaggtcaccttccgaaagcctaccttctgcgacagctgcagtggcttcctctggggtgtcaccaagcaaggctaccgctgtcgggagtgcgggctgtgttgccacaaacactgcagagaccaggtgaaggtagaatgtaagaagaggccaggggccaagggcgatgcaggaccccccggagctcctgtcccatccacaccagctccccatgccagctgtggctccgaggaaaatcactcctacacgctatccctggagcctgagactgggtgccagcttcgccatgcctggacccagactgaatccccacacccttcctgggaaacagatacggtcccctgcccggtgatggacccaccatcaactgcatcctccaagctggattcctag

3-5

Atgaacagaaaagacagtaagaggaagtcccaccaggaatgcaccggaaaaacaggagggcgaggccggccccgccaagtgcgccgccacaagacatgccccagccctcgggaaatcagcaaggtcatggcttccatgaacctgggcctgctgagtgagggcggctgcagcgaagatgagctgctggagaaatgcatccagtccttcgattcagctggcagcctgtgccacgaggaccacatgctcaacatggtgctggccatgcacagctgggtgctgccgtccgccgacctggctgcccgcctgctgacctcataccagaaggccacaggggacacccaggagctgagacggctgcagatctgtcacctggtcaggtactggctgatgcgacaccctgaggtgatgcaccaggatccccagctagaagaagtcataggtcgtttctgggccaccgtggcccgggagggcaactcagcccagagaagactgggagactcttctgacctcccaggcctgggcaaaaagcgcaaagtgtccttgcttttcgaccacttggagacgggggagctggctcagcacctcacctacctggagttccggtccttccaggctatcacgccccaggacctgcggagctacgttttgcagggctcagtacgaggctgcccggccctggagggctccgtaggtctcagcaacagcgtgtcccgctgggtgcaggtgatggtgctgagccgtcccgggcccctacagcgtgcacaggtgctggacaagttcattcacgtggcacagaggctccaccagctgcagaatttcaacacgctgatggcagtcacagggggcctgtgtcacagtgccatctccagactcaaggactcccatgcccacctgagccctgacagcaccaaggccctcctggagctcactgagctccttgcctcccacaacaactacgcccgctaccgccgcacctgggctggctgcgcgggtttccggctgcctgtactgggcgtgcacctcaaggacctggtgtccctgcatgaggcacagcccgacaggttgcctgacggccgcctgcacctacccaagctgaacaacctctacctgcggctgcaggagctggtggccctccaagggcagcatccaccctgcagcgccaatgaggatctgctgcacctgctcacgctctccctggacctcttctacacggaagacgagatctatgagctttcttatgcccgggagccgcgttgtcccaagagcctgccaccctcccccttcaatgcacctctggtggtggagtgggcccctggtgtgacacccaagccggacagggtcacactgggtcggcatgtggagcagctggtggagtctgtgttcaagaattatgaccctgaaggccgaggaacaatctctcaggaggactttgagcgactctcgggcaattttcccttcgcctgccatgggcttcacccacccccacgccaggggagaggatccttcagcagagaggagctgacagggtacctgctccgggccagcgccatctgctccaagttgggcctggccttcctgcacaccttccatgaggtcaccttccgaaagcctaccttctgcgacagctgcagtggcttcctctggggtgtcaccaagcaaggctaccgctgtcgggagtgcgggctgtgttgccacaaacactgcagagaccaggtgaaggtagaatgtaagaagaggccaggggccaagggcgatgcaggaccccccggagctcctgtcccatccacaccagctccccatgccagctgtggctccgaggaaaatcactcctacacgctatccctggagcctgagactgggtgccagcttcgccatgcctggacccagactgaatccccacacccttcctgggaaacagatacggtcccctgcccggtgatggacccaccatcaactgcatcctccaagctggattcctag

4-4

Atgaacagaaaagacagtaagaggaagtcccaccaggaatgcaccggaaaaacaggagggcgaggccggccccgccaagtgcgccgccacaagacatgccccagccctcgggaaatcagcaaggtcatggcttccatgaacctgggcctgctgagtgagggcggctgcagcgaagatgagctgctggagaaatgcatccagtccttcgattcagctggcagcctgtgccacgaggaccacatgctcaacatggtgctggccatgcacagctgggtgctgccgtccgccgacctggctgcccgcctgctgacctcataccagaaggccacaggggacacccaggagctgagacggctgcagatctgtcacctggtcaggtactggctgatgcgacaccctgaggtgatgcaccaggatccccagctagaagaagtcataggtcgtttctgggccaccgtggcccgggagggcaactcagcccagagaagactgggagactcttctgacctcccaggcctgggcaaaaagcgcaaagtgtccttgcttttcgaccacttggagacgggggagctggctcagcacctcacctacctggagttccggtccttccaggctatcacgccccaggacctgcggagctacgttttgcagggctcagtacgaggctgcccggccctggagggctccgtaggtctcagcaacagcgtgtcccgctgggtgcaggtgatggtgctgagccgtcccgggcccctacagcgtgcacaggtgctggacaagttcattcacgtggcacagaggctccaccagctgcagaatttcaacacgctgatggcagtcacagggggcctgtgtcacagtgccatctccagactcaaggactcccatgcccacctgagccctgacagcaccaaggccctcctggagctcactgagctccttgcctcccacaacaactacgcccgctaccgccgcacctgggctggctgcgcgggtttccggctgcctgtactgggcgtgcacctcaaggacctggtgtccctgcatgaggcacagcccgacaggttgcctgacggccgcctgcacctacccaagctgaacaacctctacctgcggctgcaggagctggtggccctccaagggcagcatccaccctgcagcgccaatgaggatctgctgcacctgctcacgctctccctggacctcttctacacggaagacgagatctatgagctttcttatgcccgggagccgcgttgtcccaagagcctgccaccctcccccttcaatgcacctctggtggtggagtgggcccctggtgtgacacccaagccggacagggtcacactgggtcggcatgtggagcagctggtggagtctgtgttcaagaattatgaccctgaaggccgaggaacaatctctcaggaggactttgagcgactctcgggcaattttcccttcgcctgccatgggcttcacccacccccacgccaggggagaggatccttcagcagagaggagctgacagggtacctgctccgggccagcgccatctgctccaagttgggcctggccttcctgcacaccttccatgaggtcaccttccgaaagcctaccttctgcgacagctgcagtggcttcctctggggtgtcaccaagcaaggctaccgctgtcgggagtgcgggctgtgttgccacaaacactgcagagaccaggtgaaggtagaatgtaagaagaggccaggggccaagggcgatgcaggaccccccggagctcctgtcccatccacaccagctccccatgccagctgtggctccgaggaaaatcactcctacacgctatccctggagcctgagactgggtgccagcttcgccatgcctggacccagactgaatccccacacccttcctgggaaacagatacggtcccctgcccggtgatggacccaccatcaactgcatcctccaagctggattcctag
